# Reservoir Computing Enabled by Polymer Electrolyte-Gated MoS_2_ Transistors for Time-Series Processing

**DOI:** 10.3390/polym17091178

**Published:** 2025-04-25

**Authors:** Xiang Wan, Qiujie Yuan, Lianze Sun, Kunfang Chen, Dongyoon Khim, Zhongzhong Luo

**Affiliations:** 1 College of Integrated Circuit Science and Engineering, Nanjing University of Posts and Telecommunications, Nanjing 210023, China; wanxiang@njupt.edu.cn (X.W.); 1023223314@njupt.edu.cn (Q.Y.); 1023223313@njupt.edu.cn (K.C.); 2 College of Electronic and Optical Engineering, Nanjing University of Posts and Telecommunications, Nanjing 210023, China; 1023020628@njupt.edu.cn

**Keywords:** reservoir computing, polymer electrolyte, MoS_2_

## Abstract

This study presented a novel reservoir computing (RC) system based on polymer electrolyte-gated MoS_2_ transistors. The proposed transistors operate through lithium ion (Li^+^) intercalation, which induces reversible phase transitions between semiconducting 2H and metallic 1T’ phases in MoS_2_ films. This mechanism enables dynamic conductance modulation with inherent nonlinearity and fading memory effects, rendering these transistors particularly suitable as reservoir nodes. Our RC implementation leverages time-multiplexed virtual nodes to reduce physical component requirements while maintaining rich temporal dynamics. Testing on a spoken digit recognition task using the NIST TI-46 dataset demonstrated 95.1% accuracy, while chaotic time-series prediction of the Lorenz system achieved a normalized root mean square error as low as 0.04. This work established polymer electrolyte-gated MoS_2_ transistors as promising building blocks for efficient RC systems capable of processing complex temporal patterns, offering enhanced scalability, and practical applicability in neuromorphic computation.

## 1. Introduction

Reservoir computing (RC) represents an innovative and computationally efficient paradigm derived from recurrent neural networks (RNNs) [1]. Unlike traditional RNNs, RC simplifies the training process by exclusively optimizing the weights of the output layer, thereby circumventing the need for resource-intensive full-network optimization techniques such as backpropagation. This approach significantly reduces computational overhead while maintaining robust performance. The low computational demand of RC makes it suitable for edge computing, especially in systems where it can quickly respond to physical signals and adapt to dynamic scenarios [2]. RC is inherently suited for time-series tasks by leveraging the memory properties of the reservoir to capture historical dependencies in the input. Most of the weights in the RC system are fixed, eliminating the need to adjust internal connections, which also reduces the risk of overfitting.

Various nanoscale devices [3] have been employed as the dynamic nonlinear nodes in RC system including memristive [4,5], spintronic [6], ferroelectric [7], and memcapacitive devices [8]. The physical characteristics of these devices inherently exhibit nonlinearity and fading memory effects. Nonlinear mapping of input–output relationships is essential for effectively separating input features within a high-dimensional space, enabling complex tasks like classification or prediction [9]. Fading memory, on the other hand, describes the gradual decay of the system’s dependence on past inputs, reflected in the temporal evolution of its internal states. This property is vital for processing temporal data (e.g., speech, chaotic sequence), where only recent information is relevant [10]. Time-division multiplexing splits the gradual delay into discrete intervals, creating “virtual nodes” that retain transient responses to recent inputs. Two-terminal devices was predominant in RC studies due to their inherent simplicity, scalability, and compatibility with neuromorphic hardware architectures [11,12]. However, they present challenges because they merge input and read operations into a single terminal, causing interference in the capture of reservoir states, particularly when using time-multiplexed virtual nodes. These nodes are “sampled” sequentially via the read operation to capture the transient state of the nonlinear device. In two-terminal devices, however, the read signal itself has an impact on the device’s internal state (e.g., altering its resistance).

Ion-conducting polymer electrolytes present a fascinating avenue for implementing reservoir nodes in the RC system. Ionic conduction and interactions often create nonlinear behavior under applied voltage. The ionic response to voltage input exhibits a fading memory effect due to the relaxation dynamics of the ions and their transient interactions, which decay over time. This creates a temporal memory window where past inputs continue to influence the system’s current state. In this work, we proposed a RC system enabled by polymer electrolyte-gated MoS_2_ transistors. The MoS_2_ channel undergoes reversible phase transitions via lithium–ion (Li^+^) intercalation from the polymer electrolyte under gate voltage. By exploiting the transient ionic interactions and phase-dependent conductance, the transistor-based physical reservoir node captures historical input dependencies while minimizing hardware complexity. The proposed RC system was demonstrated through spoken digit recognition and prediction of the chaotic system. This work advances the development of edge-compatible RC systems, offering insights into material-driven neuromorphic engineering for next-generation machine learning applications.

## 2. Materials and Methods

### 2.1. Materials

Lithium perchlorate (LiClO_4_, 99.9% metals basis) was obtained from Shanghai Aladdin Biochemical Technology Co., Ltd., Shanghai, China. Poly (methyl methacrylate) (PMMA, 98%) was obtained from Energy Chemical, Shanghai, China. Ethylene carbonate (EC, 98%) was purchased from Shanghai Macklin Biochemical Co., Ltd., Shanghai, China. Propylene carbonate (PC, 98%) and ethyl lactate (≥98%) were obtained from Sigma-Aldrich (Shanghai) Trading Co., Ltd., Shanghai, China in their as-received states.

### 2.2. Synthesis of Polymer Electrolyte Solution

PMMA solution (20 wt%) was prepared by dissolving 1 g of PMMA in 5 mL of ethyl lactate using magnetic stirring (500 rpm) combined with ultrasonic treatment (40 kHz, 25 °C) for 4 h. A mixture of 125 mg of LiClO_4_, 1250 mg of EC, and 1250 μL of PC was magnetically stirred (300 rpm) and ultrasonicated (40 kHz, 25 °C) for 2 h to obtain lithium salt solution. The PMMA and lithium salt solutions were then homogenized via continuous magnetic stirring to form the final ion-conducting polymer electrolyte.

### 2.3. Device Fabrication

**Figure 1:**
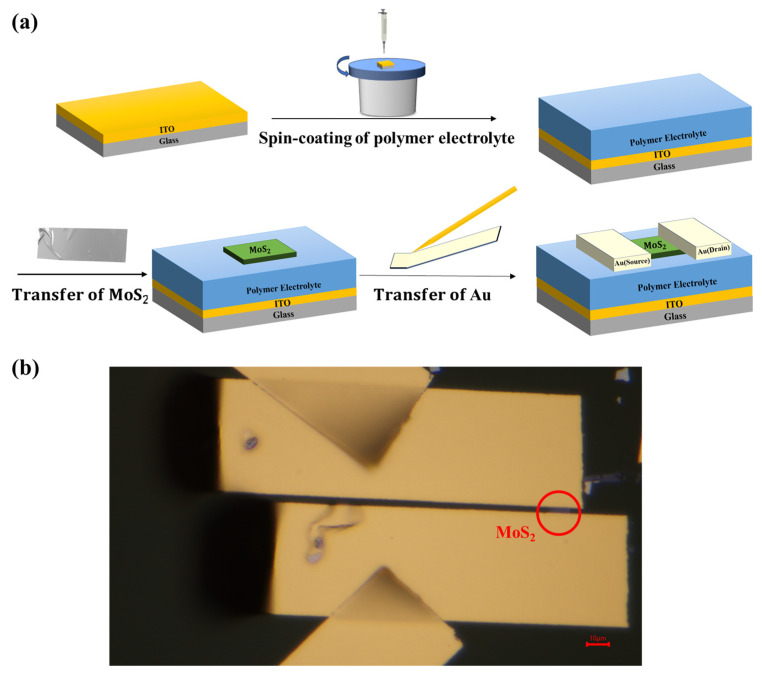
(**a**) Fabrication process and (**b**) top-view optical microscopic image of the polymer electrolyte-gated MoS_2_ transistor.

The fabrication process of the polymer electrolyte-gated MoS_2_ transistor is shown in Figure 1a. The polymer electrolyte solution was spin-coated onto pre-cleaned ITO glass substrates under a nitrogen atmosphere with a two-step profile of 500 rpm for 10 s, followed by 4000 rpm for 60 s. After spin-coating, a stepwise annealing process was conducted to form the dielectric layer, consisting of annealing at 50 °C for 5 min and then at 80 °C for 1 h. A MoS_2_ flake was obtained and transferred onto the dielectric layer via mechanical exfoliation. Transferred van der Waals metal electrodes (150 nm thick Au) were used as source and drain. The optical microscopic image of the device is shown in Figure 1b.

### 2.4. Measurements and Simulations

The electrical characteristics of the polymer electrolyte-gated MoS_2_ transistors were measured using a Keysight B1500 Semiconductor Precision Analyzer by Keysight Technologies, Santa Rosa, CA, USA. The simulations were carried out in Python 3.12.

## 3. Results

### 3.1. Operation Mechanism

**Figure 2:**
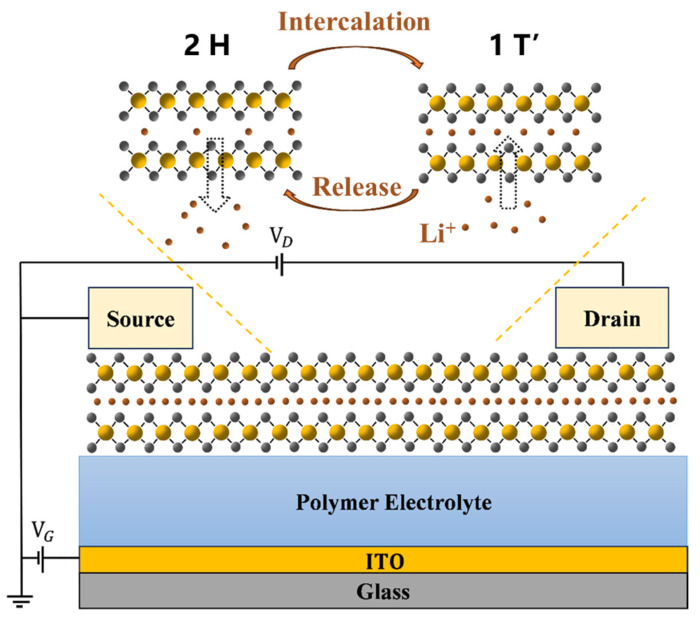
Reversible phase transition in MoS_2_ films induced by Li^+^ intercalation from the polymer electrolyte, which is facilitated by the gate voltage.

MoS_2_ can naturally exist in two distinct phases: the semiconducting 2H phase and the metallic 1T’ phase [13,14]. The conductance of MoS_2_ film can be dynamically modulated by inducing a phase transition between these two phases through the intercalation of Li^+^ into the MoS_2_ lattice [15,16]. In the as-fabricated polymer electrolyte-gated MoS_2_ transistors, the layered MoS_2_ films are particularly well suited to accommodate the reversible storage and release of Li^+^ [17]. An applied gate-to-channel electric field can cause the ions from the polymer electrolyte to be intercalated into the MoS_2_ lattice, thus altering the local electronic conductance of the material, as shown in Figure 2. The reversible 2H ⟶ 1T’ phase transition in MoS_2_, driven by Li^+^ intercalation under gate voltage, is a well-established mechanism supported by experimental evidence [18]. Li^+^ intercalation suppresses the 2H-phase while introducing the distinct 1T’-phase. XPS analysis confirms a ~0.7 eV reduction in Mo 3d binding energy due to electron transfer from Li to Mo, stabilizing the metallic 1T’ phase [19]. This phase transition occurs via shear-driven layer sliding, forming dislocation-decorated phase boundaries during Li^+^ redistribution. These structural and electronic changes align with hysteresis and reversible memory behavior, as will be shown in the subsequent sections.

### 3.2. Electrical Characteristics

**Figure 3:**
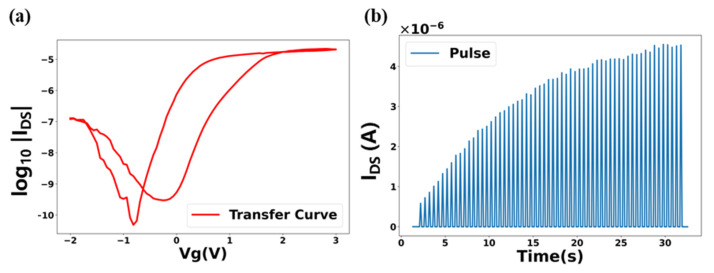
(**a**) Transfer curve and (**b**) short-term potentiation of current spikes of polymer electrolyte-gated MoS_2_ transistor.

Figure 3a shows the transfer characteristic of the device under gate voltage sweep. At lower gate voltages, the MoS_2_ film is predominantly in the 2H semiconducting phase, which exhibits low device conductance. As the gate voltage increases, Li^+^ start intercalating into the MoS_2_ lattice. This results in a transition from the 2H phase to the 1T’ metallic phase, sharply increasing the device conductance. At higher gate voltages, the film saturates into the 1T’ phase. The conductance levels of most available sites within the MoS_2_ have been intercalated with Li^+^ ions. As the gate voltage is reduced, the I–V curve shows hysteresis. This is because the Li^+^ remain intercalated longer than when they were introduced, leading to a delay in the reversal of the phase transition. Eventually, the Li^+^ de-intercalate sufficiently, and the MoS_2_ film reverts primarily to the 2H phase, returning to its initial conductance at a lower gate voltage. Figure 3b shows short-term potentiation of current spikes in response to multiple gate voltage pulses, from −0.5 V (base) to +0.5 V (peak). The MoS_2_ film starts at a baseline conductance, corresponding to a mostly 2H phase in the absence of prior stimulus. Applied gate voltage pulses cause a temporary increase in Li^+^ intercalation, which enhances device conductance. The impact of each pulse fades over time, exhibiting short-term memory until natural diffusion resets the MoS_2_ film. With successive gate voltage pulses, the MoS_2_ film experiences cumulative intercalation, leading to a gradual increase in the device’s current response. Such a fading memory effect, where history of recent stimulation-induced Li^+^ intercalation influences immediate conductance state, endows the device with context sensitivity—the ability to “remember” previous inputs over short durations. This characteristic is vital for emulating the reservoir nodes in the RC system, particularly for handling time-series data that requires contextual awareness.

### 3.3. RC System Based on Polymer Electrolyte-Gated MoS_2_ Transistors

**Figure 4:**
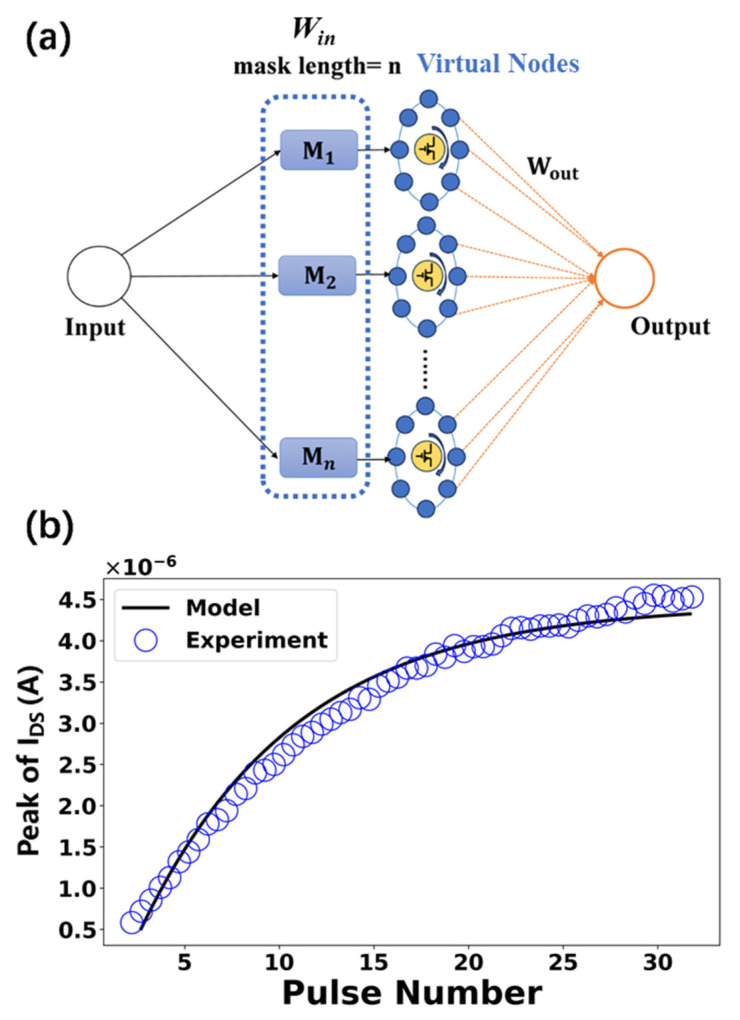
(**a**) Structure of the RC system based on the polymer electrolyte-gated MoS_2_ transistors; (**b**) fitting results comparing experimental data and simulation for the short-term potentiation of current peaks.

The RC system based on the polymer electrolyte-gated MoS_2_ transistors operates with a simplified structure, which is shown in Figure 4a. The input time-series data of each time step, whether single-dimensional or multi-dimensional, is first encoded with values ranging from 0 to 1, which are proportional to duty cycles of the applied gate voltage pulses. The encoding is linearly transformed through a random and fixed input weight network (mask) *W_in_*. The device functions as the reservoir. When the gate voltage pulse is applied, Li^+^ migration causes a transient change in device conductance accordingly. The transient increase in the device current during the gate voltage is modeled by

(1) $$\;\;I=(I_{inf}-I)\cdot \left[1-exp\left(-\frac{t}{\tau_{1}}\right)\right]$$

The transient decrease in the device current without the gate voltage is modeled by

(2) $$\;I=(I-I_{ini})\cdot exp\left[-{\left(\frac{t}{\tau_{2}}\right)}^{\beta }\right]$$

The parameters are set as follows: The time constant for ionic accumulation, *τ*_1_, is 4.7. The time constant for ionic relaxation process, *τ*_2_, is 3.3, and the stretch index, β, is 1.55. The limitations of current increase and decrease, *I_inf_* and *I_ini_*, are 10 μA and 0.1 nA, respectively. Figure 4b shows the fitting results for the short-term potentiation of current peaks at a constant source–drain voltage of 1.0 V. This model was employed in simulating the RC system, wherein the sampled device current is recorded as the reservoir state. The sampled current embodies a fading memory effect, recalling recent inputs while gradually forgetting older ones. This ability to recollect past inputs is foundational to RC systems, as it inherently encodes temporal dependencies within time-series data. The history of inputs is implicitly encoded in the device’s transient conductance response, which ensures that older inputs progressively decay in influence while newer inputs dominate, forming a time-weighted representation of sequential features. This is also beneficial for applications such as predicting future events and recognizing time-series patterns. During each time step, multiple states (virtual nodes) of each reservoir can be obtained by increasing the sampling rate. The reservoir states (current values) are linearly processed through the output weighting network *W_out_*, where the weights are adjusted during the training process to generate the desired output. Utilizing the inherent nonlinearity and fading memory of the polymer electrolyte-gated MoS_2_ transistors provides rich temporal dynamics necessary for handling complex time-series data. The utilization of such physical reservoirs and virtual nodes can significantly reduce the number of physical components traditionally required for RC systems with a large number of interconnected nodes. This enhances the scalability of the system and makes it more suitable for practical applications.

### 3.4. RC System for Spoken Digit Recognition

**Figure 5:**
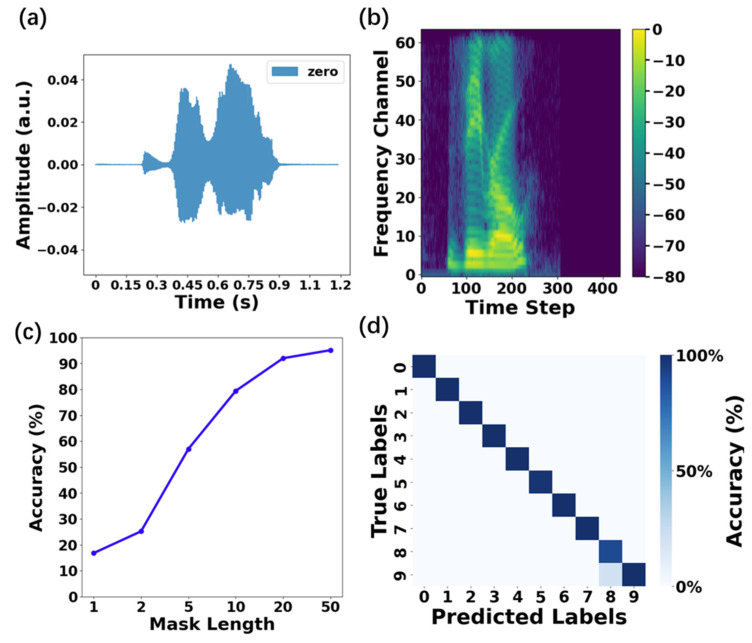
(**a**) Audio waveform and (**b**) magnitude spectrum in dB scale corresponding to a spoken digit zero in the dataset; (**c**) the recognition accuracy as a function of mask length; (**d**) the confusion matrix of the recognition for a mask length of 50.

The RC system based on polymer electrolyte-gated MoS_2_ transistors was then demonstrated on a spoken digit recognition task using the NIST TI-46 dataset. This dataset comprises isolated audio waveforms of English digits (0–9) spoken by five female speakers. The objective is to classify digits independently of speaker identity. The raw audio waveforms (resampled at 8 kHz, Figure 5a) are preprocessed using short-time Fourier transform (STFT) to decompose the temporal signal into a time–frequency representation, as shown in the magnitude spectrum in dB scale (Figure 5b). Each audio frame is filtered into 64 frequency channels, resulting in a 64-dimensional time-series sequence. The sequence was used as the input data and linearly transformed via a fixed random input weighting matrix (*W_in_*) and normalized to a [0, 1] range. These normalized values are encoded as duty cycles of gate voltage pulses applied to the polymer electrolyte-gated MoS_2_ transistor, which serves as the reservoir. The device current responds dynamically to the input pulses, leveraging its inherent nonlinearity and fading memory to map temporal dependencies in the spectral sequences. The reservoir state—sampled as the device current at the end of each pulse—encodes a transient memory of recent spectral inputs, enabling temporal feature extraction. The dataset (500 audio samples) is partitioned into 450 training and 50 testing samples. During training, the output weights (*W_out_*) are optimized via ridge regression to minimize the mean squared error (MSE) between the predicted and true one-hot encoded labels (10-dimensional vectors representing digit classes 0–9). For testing, the output node with the largest value determines the predicted digit class. Systematic evaluation via 10 repeated simulations revealed a peak average accuracy of 95.1% at a mask length of 50 (Figure 5c). The confusion matrix analysis for a mask length of 50 (Figure 5d) confirmed robust speaker-independent recognition, with minimal misalignment between predicted and true digit labels.

### 3.5. RC System for Chaotic Time-Series Prediction

**Figure 6:**
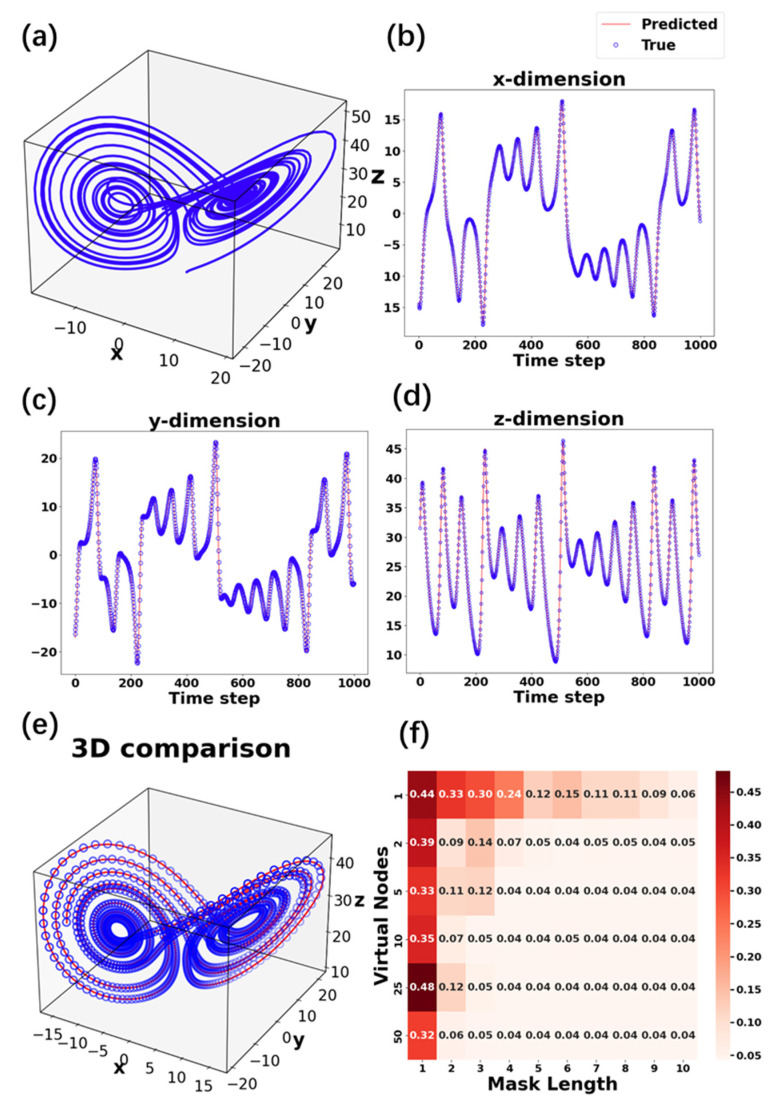
(**a**) Actual trajectory of the Lorenz system (3000 data points, including training and test datasets); (**b**) the *x*-, (**c**) *y*-, (**d**) *z*-, and (**e**) three-dimensional display of the predicted and actual Lorenz trajectories for the test dataset (last 1000 data points), with 5 virtual nodes and mask length of 4; (**f**) NRMSE of the prediction as a function of the number of virtual nodes and mask length.

The RC system based on polymer electrolyte-gated MoS_2_ transistors was subsequently employed to solve a chaotic time-series prediction task, specifically the Lorenz system. The Lorenz system is a canonical model of atmospheric convection, renowned for its nonlinear, deterministic, and chaotic behavior. The Lorenz system is defined by

(3) $$\frac{dx}{dt}=\sigma \left(y-x\right),$$

(4) $$\frac{dy}{dt}=x\left(\rho -z\right)-y,$$

(5) $$\frac{dz}{dt}=xy-\beta z,$$

with parameters *σ* = 10, *ρ* = 28, and *β* = 8/3. The Lorenz equations were numerically solved using the fourth-order Runge–Kutta method with a fixed time step (Δ*t* = 0.01) and initial values (*x*_0_ = 1, *y*_0_ = 1, *z*_0_ = 1). These parameters induce chaotic dynamics, producing the iconic “butterfly” attractor, as shown in Figure 6a. The following task involves predicting the future state (*x_n_*_+1_, *y_n_*_+1_, *z_n_*_+1_) from the current input (*x_n_*, *y_n_*, *z_n_*), testing the RC system’s ability to model complex temporal dependencies. The three-dimensional chaotic sequences for each time step were used as the input data and linearly transformed through a random and fixed input weighting network *W_in_*, and subsequently normalized to a range from 0 to 1 as the duty cycle of gate voltage pulses. The first 2000 data points of the chaotic sequences were used for training; the remaining 1000 data points served as test data. The output weight matrix *W_out_* was optimized using ridge regression. Figure 6d demonstrates close alignment between the predicted and actual Lorenz trajectories for each dimension, with 5 virtual nodes and mask length of 4. The three-dimensional attractor reconstructed from the RC’s predictions in Figure 6e retains the topological structure of the original Lorenz system, confirming the system’s preservation of dynamical features. The system’s prediction error (root mean square error, NRMSE), varies with two parameters: mask length and the number of virtual nodes. The heatmap analysis in Figure 6f shows that the NRMSE can reach the lowest value of 0.04 with 5 virtual nodes and mask length of 4. This low error underscores the proposed RC’s efficacy in modeling chaotic systems, enabled by the MoS_2_ transistor’s nonlinearity and fading memory.

## 4. Discussion

The polymer electrolyte-gated MoS_2_ transistors exhibit distinct advantages over existing physical reservoir technologies [20]. When compared to memristors, which suffer from read–write interference due to their two-terminal architecture, our three-terminal device decouples ion intercalation from conductance readout, ensuring stable state sampling. When compared to spintronic devices, the MoS_2_ system bypasses magnetic field dependencies, simplifies system design, and enhances robustness for edge computing and biological applications. When compared to photonic reservoirs, which rely on delicate optical setups, our solid-state device achieves comparable results on spoken digit recognition and chaotic time-series prediction while being robust, compact, and ambient-stable. Furthermore, we evaluated the performance of our system with comparison to recent advancements in physical reservoir computing. Our work achieved an accuracy of 95.1% on the NIST TI-46 spoken digit recognition task, surpassing the results for recent all-polymer organic electrochemical synaptic transistors [21] and iontronic memristors [22] in similar voice recognition tasks. For chaotic time-series prediction, our system achieved an NRMSE of 0.04, superior to the results for the stochastic diffusive Ag:SiOx memristors [23] and the perovskite-based RC systems [24]. In the next phase of our research, we will focus on improving device reliability and extending operational lifespan by implementing hybrid electrolytes fabricated through initiated chemical vapor deposition (iCVD) [25] and employing advanced in situ characterization techniques. These initiatives are designed to develop robust, endurance-optimized neuromorphic components that are well suited for practical edge computing applications.

## 5. Conclusions

This study demonstrated the successful implementation of RC systems based on polymer electrolyte-gated MoS_2_ transistors, leveraging the inherent nonlinearity and fading memory effects of such devices. The reversible Li^+^ intercalation into the MoS_2_ lattice enables dynamic phase transitions between semiconducting 2H and metallic 1T’ phases, creating an effective physical reservoir with rich temporal dynamics. The implementation of virtual nodes through time multiplexing further enhances the computational capacity of the system without requiring additional physical components. The practical efficacy of the proposed RC system was validated through two challenging tasks. In spoken digit recognition using the NIST TI-46 dataset, the system achieved a remarkable accuracy of 95.1%. In the chaotic time-series prediction task using the Lorenz system, the RC system achieved an impressively low NRMSE of 0.04. This work highlights the potential of ion-conducting polymer electrolyte in neuromorphic computing, particularly for resource-constrained edge computing applications.

## Data Availability

The raw and processed data required to reproduce these findings are available on request.
